# RACE, CAREGIVING, AND PERCEIVED DISCRIMINATION AMONG WORKING ADULTS IN THE UNITED STATES

**DOI:** 10.1093/geroni/igae098.2421

**Published:** 2024-12-31

**Authors:** Maureen Templeman, William Haley

**Affiliations:** Missouri State University, Springfield, Missouri, United States; University of South Florida, Tampa, Florida, United States

## Abstract

General and workplace discrimination have been studied extensively in the general population, with Black/AA individuals consistently reporting more discrimination than White individuals. However, the intersection of race, employment, and caregiving has rarely been studied. We applied stress process and minority stress theories to address these issues. Using data from the Midlife in the United States study, we examined perceived general discrimination (lifetime and everyday) and workplace discrimination (chronic job discrimination and work inequality) among working Black/AA caregivers (n = 50), working Black/AA non-caregivers (n = 396), working White caregivers (n = 266), and working White non-caregivers (n = 2895). We conducted a series of two-way (caregiver status, race) analyses of variance and covariance to investigate the role of perceived general and workplace discrimination in the stress process for working Black/AA and White caregivers and non-caregivers. We also conducted item analyses to explore whether some experiences of discrimination were identified as particularly common for Black/AA and White caregivers. Results indicated that working Black/AA adults reported more lifetime and everyday discrimination and work inequality than working White adults. Working caregivers also reported more lifetime discrimination than working non-caregivers. Item analyses revealed several specific experiences of discrimination that were especially common among Black/AA caregivers, including being hassled by the police. Results are consistent with prior research demonstrating high levels of discrimination experienced by Black/AA individuals, but findings that working caregivers, particularly Black/AA working caregivers face additional discrimination are troubling and deserve future research attention.

